# Extracting wavelet based neural features from human intracortical recordings for neuroprosthetics applications

**DOI:** 10.1186/s42234-018-0011-x

**Published:** 2018-07-31

**Authors:** Mingming Zhang, Michael A. Schwemmer, Jordyn E. Ting, Connor E. Majstorovic, David A. Friedenberg, Marcia A. Bockbrader, W. Jerry Mysiw, Ali R. Rezai, Nicholas V. Annetta, Chad E. Bouton, Herbert S. Bresler, Gaurav Sharma

**Affiliations:** 1 Battelle Memorial Institute, 505 King Ave, Columbus, OH 43021 USA; 2 Department of Physical Medicine and Rehabilitation, The Ohio State University, Columbus, OH 43210 USA; 3 West Virginia University School of Medicine, 1 Medical Center Dr, Morgantown, WV 26506 USA; 4 Feinstein Institute for Medical Research, Manhasset, NY 11030 USA

**Keywords:** Brain computer interface, Intracortical recordings, Mean wavelet power, Signal quality, Chronic decoding

## Abstract

**Background:**

Understanding the long-term behavior of intracortically-recorded signals is essential for improving the performance of Brain Computer Interfaces. However, few studies have systematically investigated chronic neural recordings from an implanted microelectrode array in the human brain.

**Methods:**

In this study, we show the applicability of wavelet decomposition method to extract and demonstrate the utility of long-term stable features in neural signals obtained from a microelectrode array implanted in the motor cortex of a human with tetraplegia. Wavelet decomposition was applied to the raw voltage data to generate mean wavelet power (MWP) features, which were further divided into three sub-frequency bands, low-frequency MWP (*lf*-MWP, 0–234 Hz), mid-frequency MWP (*mf*-MWP, 234 Hz–3.75 kHz) and high-frequency MWP (*hf*-MWP, >3.75 kHz). We analyzed these features using data collected from two experiments that were repeated over the course of about 3 years and compared their signal stability and decoding performance with the more standard threshold crossings, local field potentials (LFP), multi-unit activity (MUA) features obtained from the raw voltage recordings.

**Results:**

All neural features could stably track neural information for over 3 years post-implantation and were less prone to signal degradation compared to threshold crossings. Furthermore, when used as an input to support vector machine based decoding algorithms, the *mf*-MWP and MUA demonstrated significantly better performance, respectively, in classifying imagined motor tasks than using the *lf*-MWP, *hf*-MWP, LFP, or threshold crossings.

**Conclusions:**

Our results suggest that using MWP features in the appropriate frequency bands can provide an effective neural feature for brain computer interface intended for chronic applications.

**Trial registration:**

This study was approved by the U.S. Food and Drug Administration (Investigational Device Exemption) and the Ohio State University Medical Center Institutional Review Board (Columbus, Ohio). The study conformed to institutional requirements for the conduct of human subjects and was filed on ClinicalTrials.gov (Identifier NCT01997125).

**Electronic supplementary material:**

The online version of this article (10.1186/s42234-018-0011-x) contains supplementary material, which is available to authorized users.

## Background

Brain Computer Interfaces (BCIs) provide a window to the brain and can help to establish a communication port to nervous system to monitor and record neural activity. Over the last decade, tremendous progress has been made in the development of BCI technology for leveraging intracortically recorded signals for controlling neuroprosthetics. Several groups have demonstrated that intracortically-recorded signals can be decoded to extract information related to imagined movements, allowing non-human primates and paralyzed humans to control computers, electronic wheelchairs, and robotic arms (Kennedy and Bakay 1998; Chapin et al. 1999; Serruya et al. 2002; Hochberg et al. 2006). In addition, these types of signals have also been used to drive activation of either chemically paralyzed arm muscles in non-human primates (Moritz et al. 2008; Ethier et al. 2012) or, as was recently shown, the paralyzed muscles of a human with tetraplegia to restore hand or arm movements (Bouton et al. 2016; Ajiboye et al. 2017).

A typical BCI-controlled neuroprosthetic system consists of recorded neural signals, a decoding method that translates these signals into control signals, and an effector such as a computer cursor, virtual keyboard, robotic arm or a functional electrical stimulation (FES) system that converts these commands into actions (Kim et al. 2011; Collinger et al. 2013; Jarosiewicz et al. 2015; Sussillo et al. 2016; Ajiboye et al. 2017). For intracortical BCIs, neural signals are recorded directly from within the motor cortex by a surgically implanted multi electrode array (MEA) (Gilja et al. 2011). However, there is no consensus on the optimal selection of neural features extracted from the intracortically-recorded signals. There are several candidate signals that can be obtained from MEAs and used for controlling neuroprosthetics, namely, local field potential (LFP), multi-unit activity (MUA), single-unit activity (SUA) and threshold crossings (Andersen et al. 2004; Bansal et al. 2012). SUA is correlated to the activity of spiking neurons and can provide excellent spatial and temporal resolution, and is known to have long term stability issues possibly due to neuronal cell death in the immediate vicinity of the electrode tip as well as biological responses at the electrode tissue interface and electrode micromotion (Rousche and Normann 1998; Williams et al. 1999; Freire et al. 2011). In contrast, LFP activity can be more stable over time as it represents a summation of the synaptic potentials of hundreds to thousands of neurons (Scherberger et al. 2005; Scheid et al. 2013), but suffers from poor spatial resolution as multiple electrodes recording from the same neurons can pick up highly correlated signals (Stark and Abeles 2007). In contrast, MUA represents an aggregated spiking activity of a population of neurons on the order of several hundred microns away from the electrode tip and can be estimated without explicit spike detection (Stark and Abeles 2007). Previous animal studies have provided strong evidence that MUA, or signals in similar frequency range, can provide a long-term stable signal for use in BCI-controlled systems (Chestek et al. 2011; Flint et al. 2013; Sharma et al. 2015). Currently, the most commonly used method to extract MUA signal from intracortical recording is to use threshold crossing (TC) that relies of setting an arbitrary threshold and band-pass filtering to isolate signal from noise (Ethier et al. 2012; Collinger et al. 2013; Ajiboye et al. 2017).

In this study, we present details of a semi-automated feature extraction method that uses wavelet decomposition to generate neural features that we call Mean Wavelet Power (MWP). Wavelet decomposition is a signal processing technique that can provide both frequency and temporal information in a data and does not require any user defined cut-off threshold and/or (Borst and Theunissen 1999; Shalchyan et al. 2012). We applied wavelet decomposition on the raw cortical data obtained over a 3-year period from a MEA implanted in the motor cortex of a human with tetraplegia. This data processing technique helped us reduce the scale of the data from ~ 3 million voltage recordings from the MEA per second to 960 MWP features per second (with 96 features each 100 ms as the processing step was 100 ms, one feature for each channel of the array each 100 ms). To aid in analysis we further characterized the MWP features into three frequency bins namely the low frequency (*lf*-MWP 0–234 Hz), mid-frequency (*mf*-MWP, 234 Hz–3.75 kHz) and high-frequency (*hf*-MWP, >3.75 kHz). We first showed that the *mf*-MWP and MUA features are not only more stable over time but also encode higher information (i.e. correlation with virtual stimulus) compared to other neural features. We also showed that, when used as inputs to a Support Vector Machine (SVM)-based decoding algorithm, the *mf*-MWP and MUA features can result in high (>90%) classification accuracy for imagined individual hand movements, respectively. The SVM decoders when retrained with using *mf*-MWP and MUA features were also able to maintain the high overall accuracy over time, respectively. Overall, our results indicate that wavelet decomposition can be a viable alternative to MUA, spike or TC based neural feature extraction methods and that *mf*-MWP based neural features can be an optimal signal for BCI decoding - it provides another robust signal for chronic applications and contains sufficient motor information to accurately decode intended movements in a paralyzed human.

## Methods

The study participant sustained complete, non-spastic quadriplegia following a traumatic spinal cord injury (SCI) occurring 4 years prior to the beginning of the study. The participant’s International Standards for Neurological Classification of SCI neurologic level is C5 AIS A (motor complete) with zone of partial preservation to C6. He had full bilateral elbow flexion (grade 5/5), active wrist extension with radial deviation through an incomplete range of motion against gravity (grade 2/5), but no motor function below the level of C6. His sensory level is C5 on the right and C6 on the left. For more details on the participant’s injury level, please refer to Bouton et al. where his use of the BCI-FES system was first reported (Bouton et al. 2016).

### Experimental system and design

A Utah MEA with 1.5 mm electrodes (Blackrock Microsystems, Inc., Salt Lake City, Utah, United States) was implanted into the hand region of the participant’s primary motor cortex in the left hemisphere. After a 1-month recovery period of the surgery for the microelectrode array, the participant started involved in the study and was cued by a virtual hand on a computer monitor to imagine different hand movements. Intracortical signals recorded by the MEA were then digitized by a NeuroPort™ (Blackrock Microsystems, Inc). A 0.3 Hz first-order high-pass and a 7.5 kHz third-order low-pass Butterworth analogue hardware filter were applied to the data, and each of the 96 channels of the MEA were sampled at a rate of 30,000 samples per second. The digitized data were then transmitted to a personal computer where it was processed in 100 ms bins, and subsequently used to train the neural decoder (see Signal Recording, Processing and Classification section below.) For daily impedance testing, the patient cable sends a 10 nA current at 1 kHz to the electrodes.

### Task conditions

**Fig. 1:**
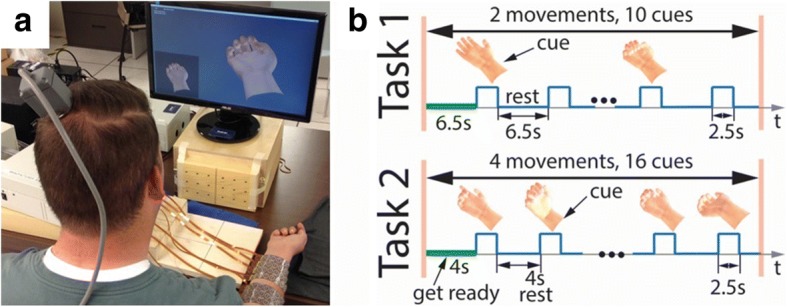
**a** System setup. The participant was seated in front of a computer monitor, where imaginary movements provided to him via a virtual hand at rest condition at the lower left corner on the screen. **b** Experimental timeline for a single block. Here, it shows a block for Task 1 and 2, respectively. In Task1, there was only two hand movements, hand opening and hand closing. While in Task2 there were four cued hand movements within each block. In between the blocks within each task, there was usually a 2–3 min break

The participant performed two distinct motor imaginary tasks at regularly spaced intervals, designated Task1 and Task2, throughout the study. In both tasks, the participant was cued by different hand-wrist movements from a virtual hand on a monitor (Fig. 1a). For Task1, a single trial of the experiment, referred to as a block, consisted of cues directing the participant to imagine hand open and hand close. There were five random repetitions of each individual movement per block and an entire block lasted about 98 s. Task2 was similar, except the participant was cued to imagine four movements: wrist flexion, wrist extension, index finger flexion and index finger extension. There were four random repetitions of each individual movement per block and each block lasted about 140 s. The participant completed two blocks per task per session. For both tasks, there was a rest period at the beginning of each block (6.5 s for Task1 and 4.5 s for Task2), followed by movement cues (duration 2.5 s) presented in a random order and separated by rest periods (6.5 s for Task1 and 4 s for Task2) (Fig. 1b). The participant was not given any feedback (visual or electrical stimulation feedback) as he attempted these movements. Not like our previous study showing online control using collected brain data (Bouton et al. 2016), the data collected in this study was from a different set of experiments in which the data was only used for off-line analysis to train a decoder and then evaluate decoding performance. During off-line analysis, the first block data within each task in each session was used to train a decoder, and the second block of data was used for testing the decoder prediction.

Each imaginary movement in Task1 was conducted 900 times in a total of 180 blocks over 90 different days throughout the course of 720 days post-implantation. Similarly, each imaginary movement in Task2 was conducted 1040 times in a total of 260 blocks over 130 different days over 3 years post-implantation.

### Signal recoding, processing and classification

Neural signals were obtained via the Utah MEA and the Neuroport™ data acquisition system. Neural data were recorded and sampled at 30 kHz. All signal processing and decoding algorithms were run on a PC using MATLAB (Release 2014a, The MathWorks, Inc., Natick, Massachusetts, United States).

#### Impedance, signal-to-noise ratio and threshold crossings measurements

**Fig. 2:**
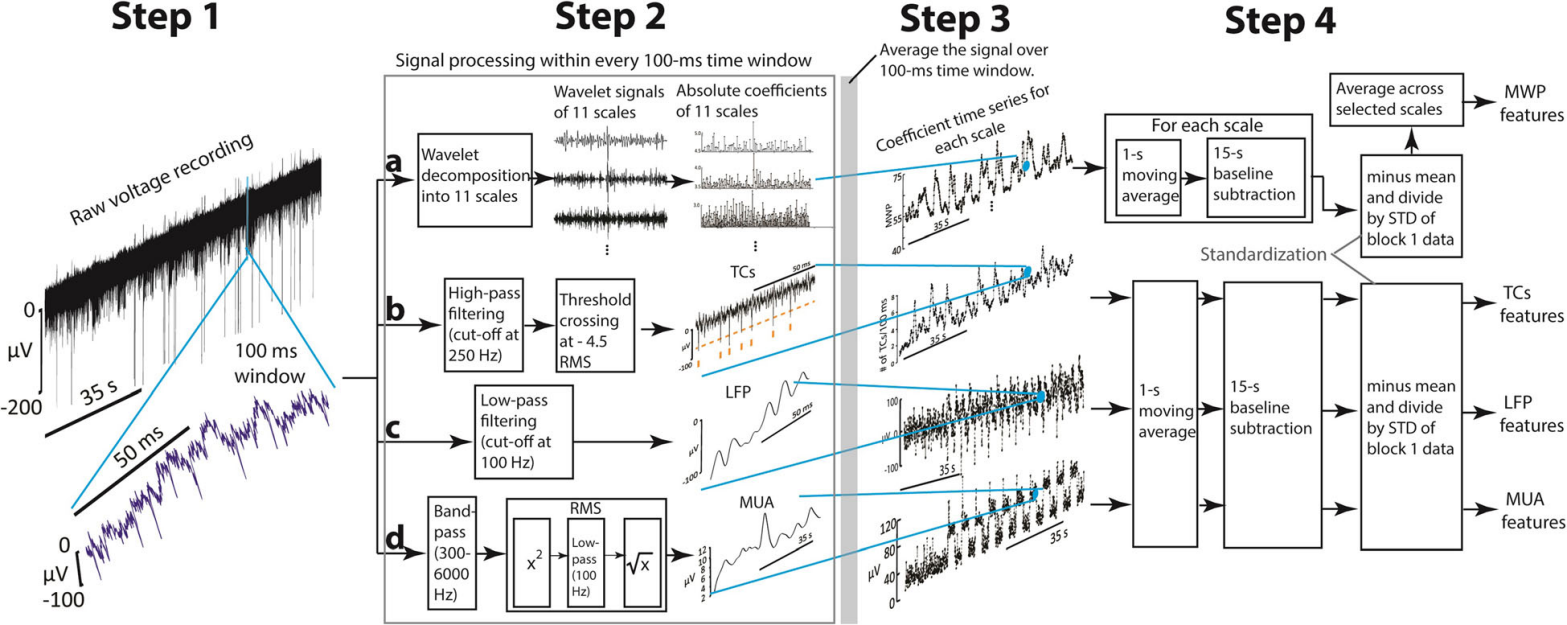
Processing of raw signal into different neural features. **Step 1**: A 100 ms section of neural signal was selected from a larger raw voltage recording. **Step 2**: Conduct signal processing for this 100 ms raw signal. In method **a**), raw signal was decomposed into 11 wavelet scales to get the rectified wavelet coefficients of each scale; In method **b**), a high-pass filter and threshold of - 4.5 times of the RMS value was applied to detect the TCs within this 100 ms section of raw signal; In method **c**), a low pass filter was applied to get LFP of the raw signal; In method **d**), band pass filter and customized RMS values were calculated to get MUA of the raw signal. **Step 3**: The processed signal within this time window were then averaged over this 100 ms, respectively, to compose the related one data point in the averaged larger time series of an entire block. **Step 4**: Signal smoothing and standardization. To generate MWP feature time series, a 1-s moving average and a 15-s wide mean subtraction were applied to this averaged time series of the entire block. Afterwards, the processed time series were standardized and averaged accordingly across selected scales to produce a new time series for each channel. To generate other feature time series, the processed signal after Step 3 was applied 1-s moving average, a 15-s mean subtraction and standardization, sequentially (Please refer to the Methods section for more details)

At the beginning of a session, the impedance of each electrode was measured at 1 kHz using Blackrock’s Impedance Tester. To compute the signal-to-noise (SNR), raw voltage recordings were first passed through a 250 Hz high pass filter. The root-mean-square (RMS) value of the noise (defined by the Blackrock Microsystems, Inc.) was then calculated from the filtered voltage traces. Next, a threshold of − 4.5 times the RMS of the noise was set to detect TCs in the voltage recording (see method b in Fig. 2) (Hochberg et al. 2012; Jin et al. 2015). The SNR for a single channel was then calculated every 2 s according to the equation, SNR $=20\times {\mathit{\log}}_{10}\frac{Signal}{Noise}$, where *signal* was the average peak-to-peak value (Hochberg et al. 2006) of the detected TCs within each two-second time window, and *noise* was the average RMS noise value of all 20-ms time windows within each two-second interval. The SNR, peak to peak value of TCs and RMS noise value for a day, were the mean of SNRs, peak to peak values, RMS noise values across all channels for all 2-s time windows recorded that day, respectively.

To generate TCs features for decoding, the total number of TCs were detected every 100 ms from the raw data to generate a TCs time series (Step 3 in Fig. 2). The TCs time series was then smoothed by applying a 1-s moving average, a 15-s baseline subtraction throughout the entire length of the time, and then standardized to generate TCs features (Step 4 in Fig. 2).

#### Wavelet decomposition and mean wavelet power

Mean wavelet power (MWP) features were computed as follows:

1. For each channel, every 100 ms of the raw voltage recording was first decomposed into 11 wavelet scales (Table 1) using the ‘db4’ mother wavelet (Farina et al. 2007). Wavelet processing was performed as described in previous study to obtain a set of wavelet coefficients (also see method a in Fig. 2) (Friedenberg et al. 2016).
2. In every 100 ms bin for each channel, the mean of the absolute coefficients for each wavelet scale was calculated to generate a mean coefficients time series for that particular channel (Step 3 in Fig. 2).
3. The mean coefficient series for each scale was then smoothed over time by applying a 1-s moving average and casual filter (average given data points before and including the current timestamp), throughout the entire time series.
4. Baseline drift was removed from the processed series within each scale by subtracting the mean value of the processed data in every 15-s time window, throughout the entire length of the time series. This procedure generated the processed mean coefficients time series of each wavelet scale for each channel.
5. a. At the end of block 1, the processed mean coefficients time series (obtained from above) for each wavelet scale were standardized by subtracting the mean and dividing by the standard deviation of each scale of block 1, respectively (Step 4 in Fig. 2). The selected scales were then averaged over each channel to generate 96 MWP features that were used as inputs to train an SVM decoder.
  b. During block 2 of the experiment the processed mean coefficients time series obtained from 4) were then standardized per channel by subtracting the mean and dividing by the standard deviation of the block 1 data (the training data) within each scale. Then, the selected scales were averaged over each channel to generate 96 MWP features (testing data) as input to the decoder.

**Table 1:** Wavelet scales and frequency bands for neural features

Wavelet scale	Frequency sub-bands (Hz)		Neural features	Frequency range
1	7500–15,000		*hf*-MWP	(Scales 1–2) > 3.75 KHz
2	3750–7500			
3	1875–3750		TCs	N/A (see Methods)
4	938–1875			
5	469–938		*mf*-MWP	(Scales 3–6) 234 Hz – 3.75 KHz
6	234–469			
7	117–234		MUA	300 Hz–6 KHz
8	59–117			
9	29–59		*lf*-MWP	(Scales 7–11) 0–234 Hz
10	15–29			
11	0–15		LFP	0 to 100 Hz

The 11 resulting scales of the standardized MWP coefficients were further classified into three sub-bands (Table 1) based on the selected power spectrum frequency ranges: high-frequency *hf*-MWP band (>3.75 kHz, scales: 1–2), with frequency range which overlaps the higher-frequency part of MUA and other high frequency signals (Stark and Abeles 2007; Perel et al. 2015); mid-frequency *mf*-MWP band (234 Hz – 3.75 kHz, scales: 3–6), with frequency range which overlaps the frequency range of MUA (Stark and Abeles 2007; Perel et al. 2015); low-frequency *lf*-MWP band (0–234 Hz, scales: 7–11), with frequency range that overlaps the LFP signals mentioned in other studies (Bansal et al. 2011; Perel et al. 2015).

#### Signal processing steps for MUA, LFP features, and tracking signal quality

To generate LFP features for decoding, in every 100 ms from the raw data, a 3rd order Butterworth low pass filter with cut off frequency at 100 Hz was applied (method c in Fig. 2). Then, the low-pass filtered signal was averaged over every 100 ms time window to generate the LFP time series. The time series was later processed by a 1-s moving average, a 15-s baseline subtraction and then standardized to generate the LFP features (Fig. 2). To generate MUA features, the raw signal was first bandpass filtered from 300 to 6000 Hz, then the customized RMS values were calculated to generate MUA signal within each 100 ms (Stark and Abeles 2007). Then, the 100-ms MUA signal was averaged across the time window and processed using the same processing steps listed above to generate MUA features (method d in Fig. 2).

To track the chronic neural signal stability over time, the neural signals were processed as follows: After getting the time series in Step 3 shown in Fig. 2, the absolute value of each neural signal time series is averaged over the entire recording time for a block of data to estimate mean strength of the signal for each channel. The mean strength of the signal is then averaged within a block for all channels, and then averaged over two blocks of data for a task to get an estimated strength of a signal for that experimental day. This calculation is repeated for all the experimental days for each signal type to generate a time series to indicate the signal strength over the course of the study. The values in the time series was then normalized relative to the first experimental day, first data point in its series, and is therefore scaled between 0 and 1 (as data shown in Fig. 5).

#### Neural signal classification

We used a nonlinear SVM with a Gaussian radial basis function, based on the open source library LIBSVM (Chang and Lin 2011), to train and classify intended movements when using the neural features as input. SVM based decoder have been shown to be one of the best performing and robust machine learning algorithms across a wide variety of datasets and fields (Fernandez-Delgado et al. 2014). In the study presented here, an individual decoder was retrained on every session day for each tested movement. These decoders were trained using features obtained from block 1 data of each task. The output of each decoder was scaled to be between − 1 and 1. The decoder with the highest output score above zero was treated as the output to indicate the predicted movement for that time point. Once trained, the performance of the decoder (s) was assessed using the data from the second experimental block. Individual movement accuracy and overall accuracy was calculated by dividing the total number of correct predictions by the total number of predictions for each movement and all movements in a task, respectively. Sensitivity for each individual movement was calculated as the ratio of correct positive predictions divided by the total number of cues for each movement. Specificity for each individual movement was calculated as the ratio of correct positive predictions for rest while the movement is not cued.

#### Calculation for spatial and temporal correlation

To investigate the effect of interelectrode distance on signal correlation, we calculated the correlation values between two possible selected channels. For a given neural feature, any two possible recording channels were selected spatially within the recording array as shown in previous study (Sharma et al. 2015). The correlation values between these paired channels were calculated using Pearson correlation, and then the absolute Pearson correlation values were arranged based on the inter-electrode distances.

To calculate the temporal correlation between neural features, absolute Pearson correlation values of any two given types of neural features from the same channel was calculated to get inter-signal correlation. Then, this process was repeated for all 96 channels to get an average correlation value of a day. The same calculation was repeated across all the investigation days to generate a temporal correlation time series for the two selected neural features (Additional file 1: Figure S1). To generate the average inter-signal correlation matrix as shown in Fig. 7, the temporal inter-signal correlation time series were averaged for each paired signal.

All statistical analysis in this manuscript were conducted using one-way analysis of variance (ANOVA) to determine whether there is any statistically significant difference between given groups of time series.

## Results

### Chronic intracortical recording quality

**Fig. 3:**
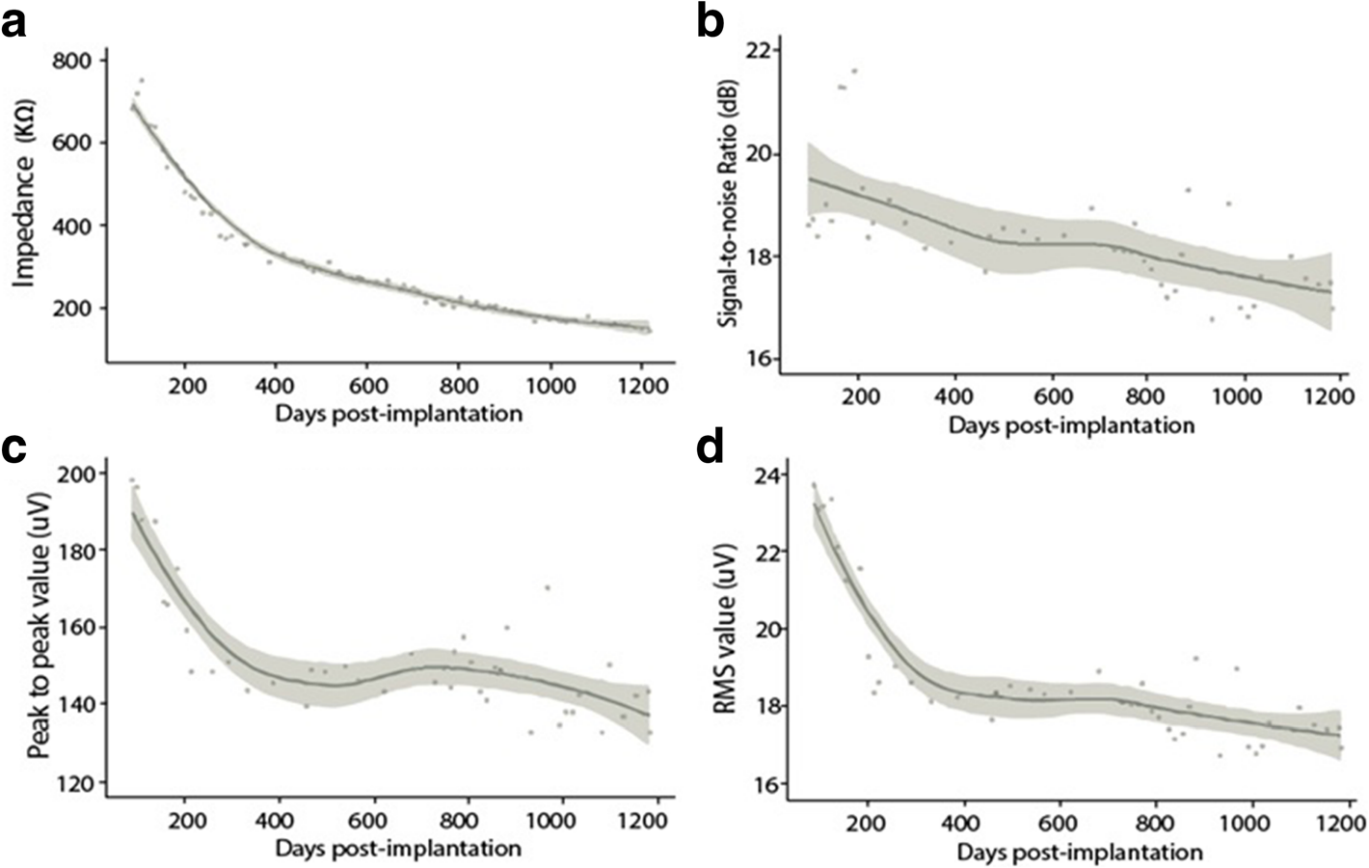
**a** Average impedance over time. Each data point here shows the average impedance across two different reference electrodes on the MEA. Most of the sharp decline happened within the first 400 days. In (**b**, **c** and **d**), the results were summarized from the data collected in Task 2. **b** Signal-to-Noise Ratio (SNR) over time. Each data point shown here is the average SNR value of an experimental day. The SNR was stable in general with an average value at 18.28 ± 0.25 dB throughout the length of the study, with a slight decline overall. **c** Peak-peak value of the detected TCs from the raw signal. Each data point presents the average peak-peak value of all the detected TCs from an experimental day. The Peak-peak value decreased the most during the first 400 days and the overall average throughout the study was 153.68 ± 17.92 μV. **d** RMS noise value of the raw signal. RMS value shared a similar temporal profile over the period of entire study compared to that of peak-peak value and SNR value. The overall average RMS noise was 18.78 ± 1.89 μV. In each figure, the line indicated the Loess regression result of the discrete values throughout the entire study, with 95% confidence intervals in each plot

To assess the quality of raw neural signals obtained from the implanted MEA, we first evaluated change in impedance and SNR values over the course of the data collection period. Average impedance of the electrodes in the MEA declined to approximately 20% of its initial value by day 1220 post-implantation (from 681.48 ± 292.8 kΩ to 140.30 ± 42.19 kΩ). Much of this decrease in impedance was during the first 400 days of the study, and the decrease was relatively small thereafter (Fig. 3a). As a quantitative measure of the reliability of the neural signals, we also analyzed the SNR of the recordings over the course of the study. SNR value dropped about 4% from an initial average of 18.58 ± 0.17 dB to 16.94 ± 0.75 dB. Overall, the SNR was 18.28 ± 0.25 dB over the course of the study (Fig. 3). This drop was also observed in the peak-to-peak value of the detected TCs and RMS noise of the raw recordings.

### The *mf*-MWP and MUA features have the clearest modulation by the virtual stimulus

**Fig. 4:**
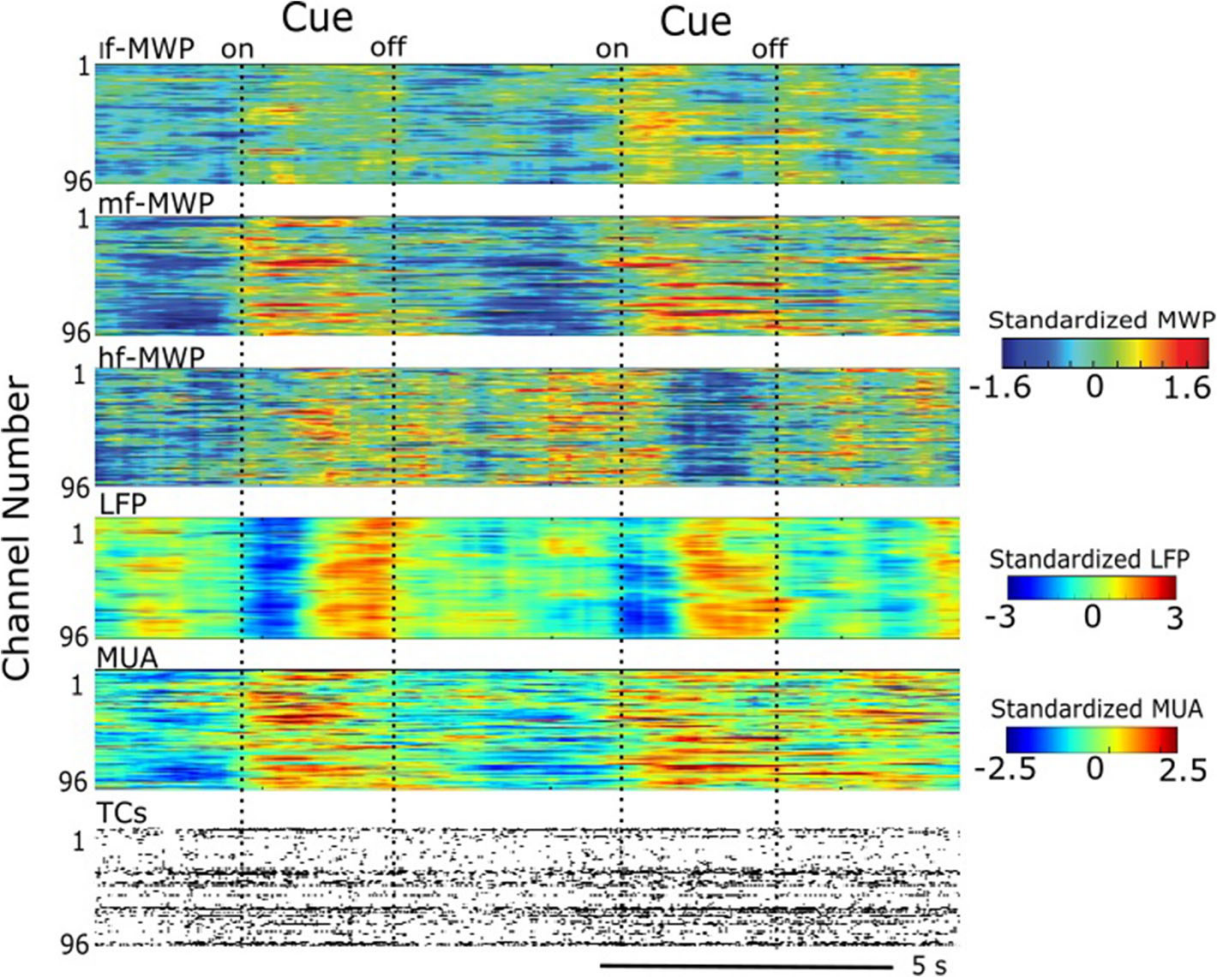
Brain signal modulated by the presentation of cues during Task 2. A representative snapshot of neural modulation as the participant imagined the cued hand movements during Task 2. Heat maps show *lf*-MWP, *mf*-MWP, *hf*-MWP, LFP and MUA features, and raster plot of TCs from raw recordings, respectively. Each time point is 100 ms. For TCs, each data point represents the total number of detected TCs within 100 ms

The heatmap in Fig. 4 shows the modulation of each type of the processed neural feature with respect to the cue (virtual stimulus). It is clear that all neural features show some degree of time-locked modulation with the cue. For the MWP-based features the *mf*-MWP feature appears to have a strong time-locked modulation compared to other MWP features.

### Using normalized signal strength as a measure of neural signal stability

**Fig. 5:**
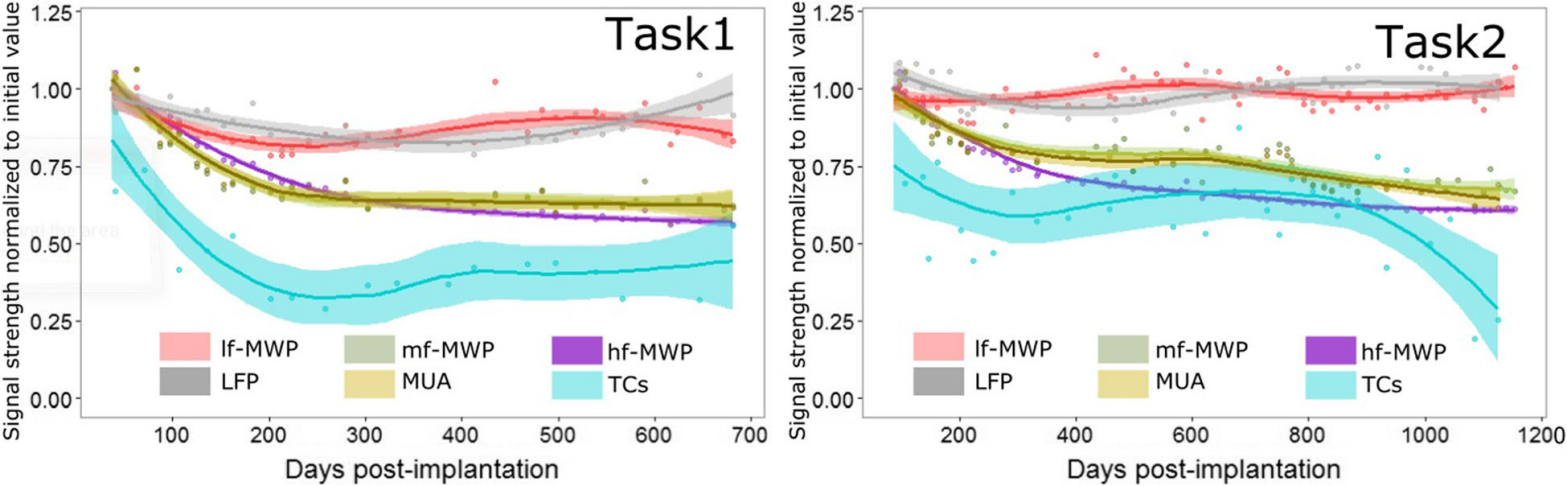
Signal stability presented by the normalized signal strength. Within each time series, the data is normalized to its initial value. In Task 1, the normalized signals strength for *mf*-MWP, *hf*-MWP, MUA and TCs were stabilized after approximately 300 days of decline, while normalized signal strength for *lf*-MWP and LFP showed a better signal stability over time. MUA and mf-MWP signals shared a very similar profile in signal declining with most of their confidence interval ranges overlap with each other. In a longer study in Task 2, similar signal stability was observed overall, while TCs signal showed a more gradual decline after 800 days post-implantation. The band in each time series shows the range of its 95% confidence interval of a LOESS fit

To further investigate whether neural signal quality changed over time, we calculated the signal strength for all the types of neural features for each MEA channel over the course of the study (See Methods). All the features were calculated from the data collected during the time when the participant performed these two tasks. We observed an initial decline in the *mf*-MWP, *hf*-MWP, and MUA signal strength over the first 300 to 400 days post-implantation, after which the neural features were relatively stable throughout the rest of the study (Fig. 5). The *lf*-MWP and LFP signal strength decreased slightly over the first 200 days post-implantation and remained relatively stable thereafter. Among these signals, the *lf*-MWP and LFP tend to have higher average normalized signal strength for both tasks, while TCs signal has the lowest average signal strength and also biggest change in signal stability over time (Fig. 5, also see Table 2).

**Table 2:** Signal strength decline in normalized features at the end of investigation (compared to their initial values)

	Task 1 (% in decline)	Task 2 (% in decline)
*mf*-MWP	40.16%	32.93%
*hf*-MWP	47.26%	38.82%
*lf*-MWP	11.25%	−7.17%
MUA	41.02%	32.01%
TCs	44.11%	74.75%
LFP	6.81%	−2.00%

Negative value in decline means the signal strength increased

### Inter-channel distance affects the level of correlation between two channels of signals

**Fig. 6:**
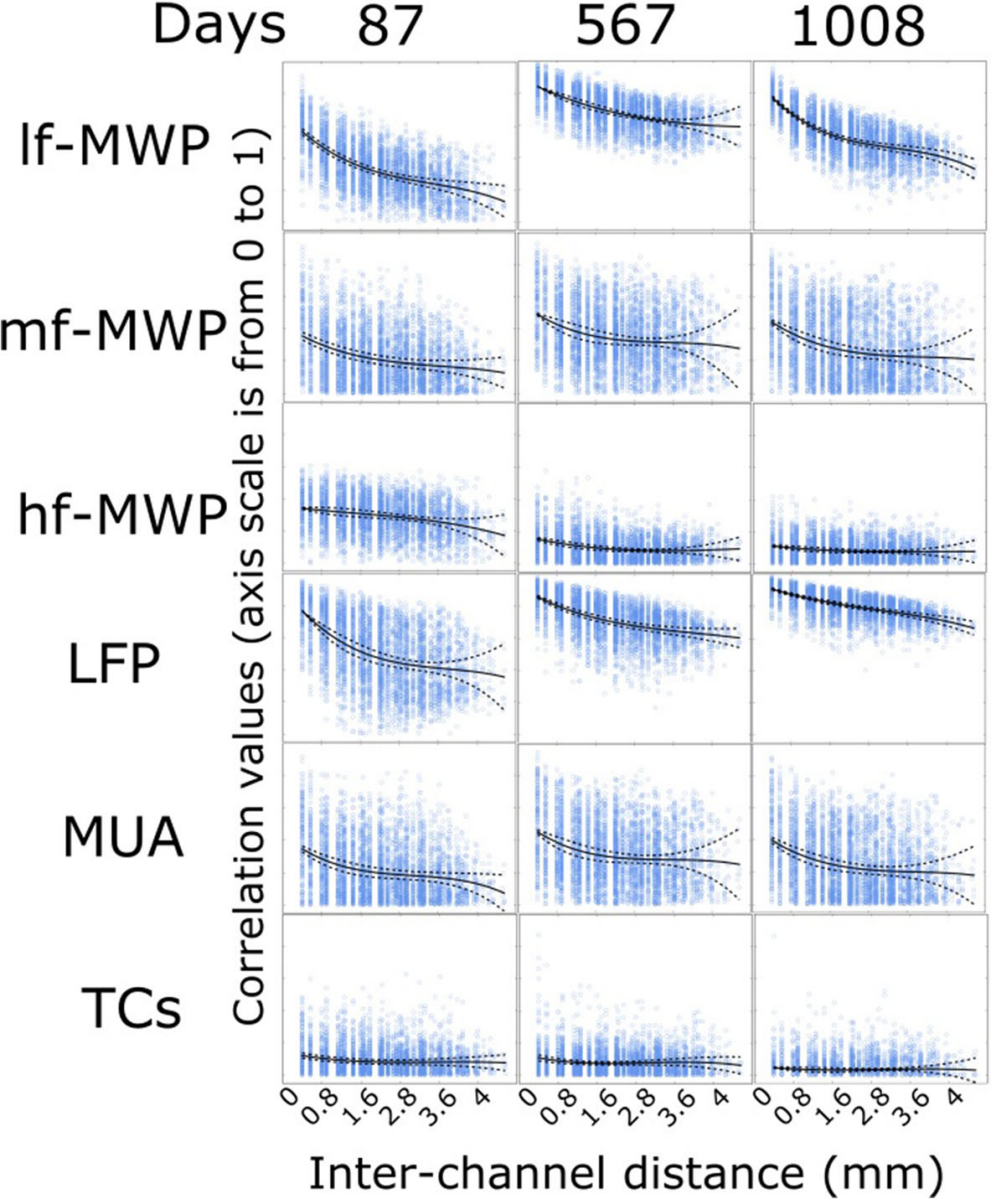
Correlation values between paired channels of neural features affected by the inter-electrode distance. We selected 3 days over the course of the study from Task 2 to investigate the effect of inter-electrode distance on neural features correlations. In each insertion, y-axis shows the correlation from 0 to 1 and x-axis shows the inter-elelctrode distance in mm. A data point represents the correlation value of two selected channels for a particular type of neural features time series. The solid line represents a 3rd polynomial fit of the data and dashed lines indicate its 95% confidence interval. In general, signal correlation decreases as the inter-elelctrode distance increases. Signal with higher frequency range has lower inter-electrode correlation, and TCs features has the lowest inter-electrode correlation values overall

We also calculated the signal correlation between two given channels for each type of neural features and investigated how the correlation changes with inter-channel distance. Our results show that, in general, there was a clear trend of decreasing correlation values as the inter-channel distance increases across all types of neural features (Fig. 6). For example, on day 87 post-implantation, *lf*-MWP features has average overall correlation values of 0.59 ± 0.01 and 0.13 ± 0.031 at inter-channel distance of 0.4 and 4.56 mm, respectively. Overall, the TCs features showed has the lowest inter-channel correlation, with values from 0.13 ± 0.01 to 0.05 ± 0.015 at 0.4 and 4.56 mm respectively, while the LFP feature showed the highest overall inter-channel correlation where the correlation values decreased from 0.91 ± 0.00 to 0.67 ± 0.02 at inter-channel distance of 0.4 and 4.56 mm, respectively (Fig. 6).

### The *mf*-MWP and MUA features showed a high level of temporal correlation

**Fig. 7:**
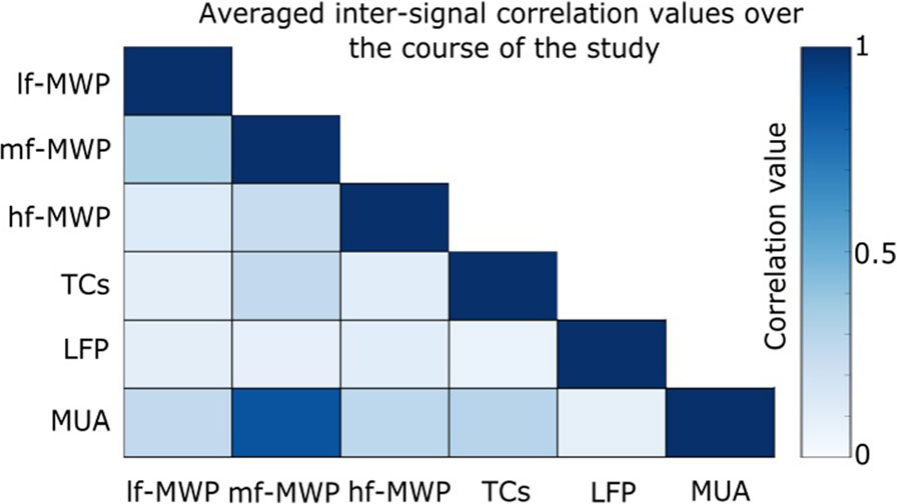
Averaged inter-signal correlation matrix. Inter-signal temporal correlation time series were averaged to give an overall correlation value for a given paired signal over the course of the study for Task 2 (For more details, please refer to Methods section and also Additional file 1: Figure S1). In general, MUA and *mf*-MWP features showed a very high level of correlation, with an average correlation value of 0.87 ± 0.19, compared to other paired neural features

We next investigated how different types of features correlated with each other over the course of the entire study. Any two possible combination among the six types of neural features were selected and computed to get average inter-signal correlation for each session day (see Methods). Among all the paired neural features, *mf*-MWP and MUA has the highest level of inter-signal correlation, with an average value of 0.83 ± 0.19 over the course of the study (Fig. 7, also Additional file 1: Figure S1).

### Classification performance is highest when using *mf*-MWP and MUA as input to the decoders

**Fig. 8:**
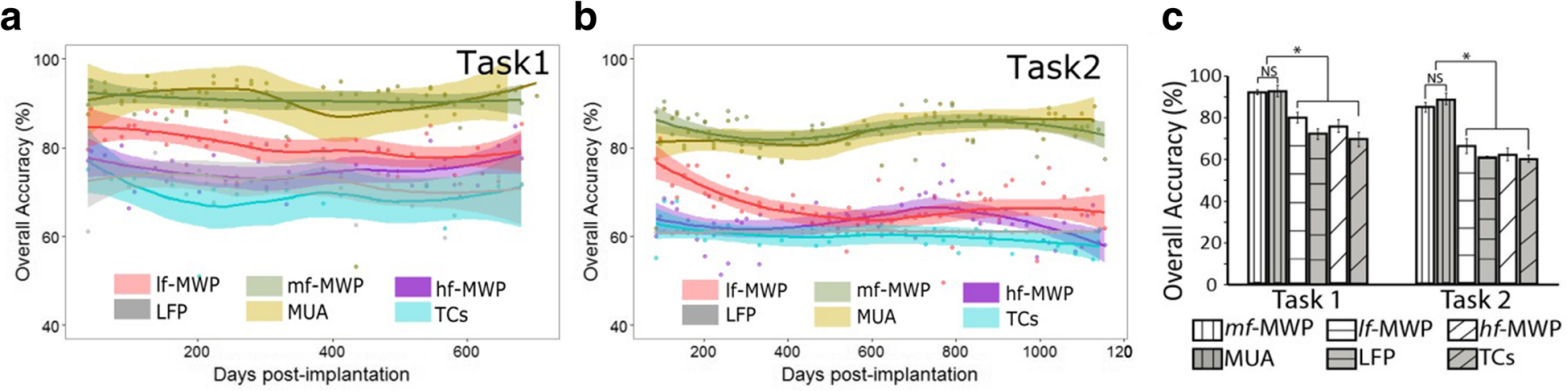
Overall classification accuracy of the decoder output when using different features as input across the entire study. **a**, **b** Each data point shows the overall accuracy from an experimental day, and the line is the LOESS regression of all the discrete data points across time. In Task 1 and Task 2, using *mf*-MWP and MUA features as input, it consistently generated the highest overall accuracy throughout the entire phase of the study (Statistical analysis indicates difference between the two overall accuracy time series were non-significant). **c** The averaged overall accuracy of the study was significantly higher (* indicates *p* < 0.001, *n* = 62 in Task1, and *n* = 64 in Task2) when using *mf*-MWP and MUA as input into the decoders, compared to those using *lf*-MWP, *hf*-MWP, LFP or TCs features as decoder input. Each error bar shows the standard deviation of the accuracy time series for a feature

We next investigated which neural feature has the richest information about the cue by looking at which neural feature could result in the most accurate decoding performance. We calculated the accuracy, sensitivity and specificity of decoding discrete imagined hand movements in Tasks 1 and 2 when each neural feature was used as inputs to the decoders. Overall, using *mf*-MWP or MUA as the decoder input resulted in consistently highest accuracy. For mf-MWP the average decoder accuracy was 91.05 ± 2.79% and 83.97 ± 3.99% for Task1 and Task2, respectively, while for MUA the average overall accuracy was 91.06 ± 6.14% and 89.44 ± 4.96% for Task 1 and Task 2, respectively (Fig. 8a, b and Table 3). Statistical analysis indicates that there is no difference between the two groups of overall accuracy in each task. Our further analysis also indicates that using MWP features with frequency ranges covering 234 Hz – 3.75 KHz (from scales 3 to 6) enables the best overall decoding accuracy with average value around 85% in Task2, while using MWP features more outside this frequency range could induce significant decrease in decoding performance (Additional file 2: Figure S2).

**Table 3:** Overall accuracy of the decoders when using different neural features as input

	Overall accuracy in Task 1 (%)	Overall accuracy in Task 2 (%)
*mf*-MWP	91.06 ± 2.79	83.97 ± 3.99
*hf*-MWP	75.33 ± 4.75	62.45 ± 4.77
*lf*-MWP	80.73 ± 4.38	66.46 ± 5.65
MUA	91.61 ± 6.14	89.44 ± 4.96
TCs	69.80 ± 5.66	60.10 ± 3.09
LFP	72.16 ± 4.94	61.24 ± 0.22

The individual accuracy for each discrete hand movement, when using *mf*-MWP and MUA as input, was always >93% within each task (Table 4). The overall accuracy metric is lower than the individual accuracy metrics because the overall accuracy accumulates errors across all the individual movements. The difference in decoding accuracy between *mf*-MWP, MUA and other neural features was even more pronounced when the participant performed Task2, which included four different discrete hand movements (compared to two movements in Task1).

**Table 4:** Accuracy of the individual decoders when using different neural features as input

	Accuracy in Task 1 (%)		Accuracy in Task 2 (%)			
	Hand open	Hand close	Wrist flexion	Wrist extension	Index flexion	Index extension
*mf*-MWP	95.34 ± 1.95	94.21 ± 2.47	96.13 ± 1.39	94.43 ± 2.16	95.14 ± 2.16	94.57 ± 1.88
*hf*-MWP	84.02 ± 3.63	85.52 ± 3.58	90.38 ± 2.75	89.90 ± 2.13	85.95 ± 2.74	86.80 ± 2.67
*lf*-MWP	87.91 ± 3.21	87.69 ± 3.81	90.19 ± 2.44	89.25 ± 2.55	88.09 ± 3.26	87.67 ± 3.36
MUA	94.95 ± 5.35	95.34 ± 3.82	96.27 ± 2.38	94.59 ± 2.31	94.38 ± 2.48	93.87 ± 2.44
TCs	86.96 ± 6.08	88.34 ± 2.46	90.99 ± 2.61	91.06 ± 0.91	90.74 ± 0.83	90.72 ± 0.68
LFP	83.22 ± 3.05	82.66 ± 3.38	85.93 ± 2.83	86.64 ± 2.54	85.18 ± 2.81	86.03 ± 2.65

Similarly, using *mf*-MWP or MUA as decoder input also resulted in higher sensitivity when compared to when *lf*-MWP, *hf*-MWP, TCs or LFP features were used as decoder inputs. (Table 5). Finally, using *mf*-MWP and MUA, respectively as decoder input resulted in higher specificity than the other neural features were used (Table 6). Overall, using *mf*-MWP or MUA feature as input to this SVM based decoder resulted in better decoding performance in classifying discrete imagined hand movements compared to other neural features.

**Table 5:** Sensitivity of the individual decoders when using different neural features as input

	Sensitivity in Task 1 (%)		Sensitivity in Task 2 (%)			
	Hand open	Hand close	Wrist flexion	Wrist extension	Index flexion	Index extension
*mf*-MWP	80.17 ± 9.36	80.45 ± 9.94	81.76 ± 9.45	74.26 ± 11.58	73.66 ± 11.53	65.98 ± 13.82
*hf*-MWP	39.17 ± 15.39	46.54 ± 17.49	38.98 ± 19.95	37.30 ± 19.94	17.97 ± 11.55	21.72 ± 12.98
*lf*-MWP	55.62 ± 15.95	56.14 ± 15.58	36.98 ± 16.99	35.64 ± 16.86	32.11 ± 16.53	33.47 ± 16.65
MUA	79.15 ± 12.51	78.80 ± 13.31	79.83 ± 12.55	71.64 ± 15.15	62.55 ± 17.12	61.36 ± 18.09
TCs	37.18 ± 5.52	30.61 ± 19.68	27.59 ± 17.16	28.00 ± 18.61	7.47 ± 9.68	12.79 ± 11.95
LFP	34.48 ± 11.08	31.34 ± 11.04	21.57 ± 9.85	31.14 ± 14.51	24.86 ± 10.16	17.40 ± 9.59

**Table 6:** Specificity of the individual decoders when using different features as input

	Specificity in Task 1 (%)		Specificity in Task 2 (%)			
	Hand open	Hand close	Wrist flexion	Wrist extension	Index flexion	Index extension
*mf*-MWP	97.82 ± 1.48	96.46 ± 2.39	97.68 ± 1.31	96.60 ± 1.82	97.45 ± 1.73	97.64 ± 1.48
*hf*-MWP	91.32 ± 4.01	91.86 ± 3.67	95.89 ± 2.58	95.55 ± 2.29	93.24 ± 2.83	93.79 ± 3.09
*lf*-MWP	93.17 ± 2.65	92.84 ± 3.35	95.90 ± 2.66	95.00 ± 2.99	94.10 ± 3.50	93.49 ± 3.49
MUA	97.51 ± 2.79	98.03 ± 2.60	98.03 ± 1.71	97.05 ± 1.60	97.80 ± 2.04	97.36 ± 1.89
TCs	94.27 ± 8.12	96.81 ± 1.84	97.24 ± 3.12	97.28 ± 2.05	98.94 ± 1.22	98.40 ± 1.13
LFP	91.16 ± 3.63	91.03 ± 3.82	92.72 ± 3.59	92.60 ± 2.73	91.65 ± 3.65	93.39 ± 3.39

## Discussion

In this study, we analyzed the chronic signal stability and decoding accuracy of different types of neural features extracted from a 96-channel MEA implanted in the motor cortex of a paralyzed human. The main findings of this paper are: 1) Electrophysiological signals are detectable from the MEA and remain highly useful for more than 3 years post-implantation; 2) Neural feature engineering using wavelet decomposition to extract MWP can provide a reliable method to analyze the spectral and temporal evolution of the brain signals, and, 3) Among the MWP-based neural features, *mf*-MWP appears to be the optimal signal for decoding imagined hand movements – it not only was robust over time but also resulted in the best decoding performance that was comparable to the performance of the other commonly used MUA feature.

During the study, there was a decline in electrode impedance, primarily occurring during the first 400 days of study, and on day 416 the average impedance dropped from 681.48 ± 292.8 kΩ to 327.29 ± 103.31 kΩ (Fig. 3a). The observed trend is consistent with previous reports where a gradual decrease in electrode impedance over time (Barrese et al. 2013; Perge et al. 2014). TCs based SNR was also measured over the course of the study to gain insights into the quality of neural signals and electrode reliability. The average SNR was 18.28 ± 0.25 dB over the course of the study (Fig. 3b). Overall, SNR value was relatively stable with a slightly decline, and there was an 8.8% decline in SNR value throughout the entire study over 3 years. The peak-to-peak value of the TCs decreased by 33.06% by day 1186 post-implantation (from 198.42 ± 8.38 μV to 132.82 ± 6.41 μV), while RMS value of the recordings decreased by 28.76% (from 23.78 ± 1.29 μV to 16.94 ± 0.75 μV) (Fig. 3c, d). The slight decrease (~ 9%) in SNR value can therefore be attributed to the bigger decrease in peak-to-peak value of the detected TCs. Similar results have been observed for the SNR of the neural signal to be relatively stable despite decrease in impedance values and amplitude of TCs over time (Barrese et al. 2013).

In this study, neural features called MWP were extracted using a custom signal processing approach based on wavelet decomposition. Wavelet transformation decomposes a time-varying signal into different frequency sub-bands while preserving its spectral and temporal characteristics (Brychta et al. 2007; Farina et al. 2007). Wavelet decomposition based features are also designed to capture important neural information and condense it into a much lower dimensional representation (Bouton et al. 2016; Sharma et al. 2016; Friedenberg et al. 2017). For example, the MWP feature extraction step reduced the size of data for each 100 ms processing bin from 288,000 raw voltage readings to 96 MWP features, one for each channel of the MEA, while retaining sufficient information to allow motor tasks related decoding (Bouton et al. 2016). This feature engineering step made it possible to process large amount of input BCI data in real-time with standard computer hardware.

Our results indicate that, qualitatively, *mf*-MWP and MUA appear to have a more robust time-locked response to the cue compared to the other type of neural features investigated (Fig. 4). The results also showed that signal strength for all neural features declined for approximately the first 200 days post-implantation (Fig. 5). This initial signal decline could be due to the initiation and resolution of inflammatory response around the electrodes and/or neuronal loss following implantation of the MEA (Biran et al. 2005; McConnell et al. 2009; Barrese et al. 2013). After the initial decline, the signal strength remained relatively stable for the duration of the study, most likely due to the stablization of glial scarring (Barrese et al. 2013; Malaga et al. 2016). Overall, the signals in the normalized *mf*-MWP, *hf*-MWP, *lf*-MWP bands declined by an average of about 40, 47, 11% respectively, while the MUA, TCs, and LFP signals declined by an average of about 41, 44, 6.81%, respectively, over 720 days post-implantation in Task1. Similar trends were observed for data collected during Task2 (Fig. 5, Table 2).

Among all neural features that we investigated, *lf*-MWP and LFP signals showed the least signal decline over the course of the study. This is not surprising as the *lf*-MWP frequency band (0–234 Hz), with power spectrum that overlaps in frequency range with that of LFP (0 to 100 Hz), represents an ensemble of synaptic activity from many neurons over a large area. Therefore, the *lf*-MWP and LFP signals are less likely to degrade due to local neurodegeneration around the electrode tips (Buzsaki 2004; Scherberger et al. 2005). These results are also in agreement with previous study, in which researchers noted that the signals in the LFP frequency band were more stable compared to higher frequency signals (Andersen et al. 2004). Normalized *mf*-MWP and MUA signals, with higher frequency range, showed a larger overall decline compared to LFP and *lf*-MWP signals in both Tasks. The *hf*-MWP signal, with frequency range similar to that of the higher frequency part of MUA and other high frequency signals, showed a significantly larger decline (*p* < 0.001, *n* = 64) compared to that of *mf*-MWP and MUA signals in Task 2. TCs signal, which is a direct detection of the the spiking neural acitivity, showed the most pronounced degradatuion in signal strength (Fig. 5). The likely cause of this decline in higher frequency signals is the potential loss of spiking neural activity near the electrode tips due to chronic, local neurodegenration (Biran et al. 2005; McConnell et al. 2009). Overall, all neural features showed a bigger initial decline compared to that in latter part of the study – a result consistent with a previous report where intracortical signal was recorded and analyzed from a non-human primate over a 7.5-month duration (Sharma et al. 2015).

It is evident from the previous discussion that the signals in the higher frequency domains (TCs, hf-MWP) degrade over time while signals in the lower frequency domains (*lf*-MWP, LFP) remain more stable. However, lower frequency signals, even those recorded from neighboring channels can be highly correlated and can therefore limit the unique information that can be extracted from these channels (Stark and Abeles 2007). Indeed, we observed that *lf*-MWP feature has the highest overall correlation within all MWP-based features and the LFP signals has the highest correlation compared to MUA and TCs signals (Fig. 6). In contrast, *mf*-MWP and MUA features are not only less correlated compared to *lf*-MWP and LFP features, but also are more stable over time compared to *hf*-MWP and TCs (Fig. 5) and therefore represent a promising robust signal to evaluate BCI decoding performance. Not surprisingly, we observed a high temporal correlation between *mf*-MWP and MUA signals (Fig. 7, also see Additional file 1: Figure S1) implying that these two features might encode similar information about the cue.

In order to investigate the information content of these neural features, we compared the use of each neural feature individually as input to an SVM-based decoder to evaluate the performance for classifying discrete imagined hand movements. While MUA and *mf*-MWP has higher correlation compare to any other paired signals, and this relationship is also reflected in the decoding results, where MUA and *mf*-MWP could achieve over 90 and 80% of decoding accuracy, respectively in Task1 and 2 (Fig. 8). When compared to other neural features, the *mf*-MWP and MUA consistently achieved and maintained a significantly higher level of decoding performance throughout the course of the study (Fig. 8). We also observed that, in general, the trend in the decoder performance over time was more stable (Fig. 8a, b), compared to the trend in the corresponding neural signal strength themselves (Fig. 5).

These results are in agreement with other studies where researchers observed that signal, with overlap in the frequency range around 300 Hz to 6 KHz, can lead to better decoder performance compared to spiking activity for classifying grasp movements in animal experiments (Stark and Abeles 2007). Some of our results are also in disagreement with findings from other groups. For example, Bansal et al., found that SUA (as calculated by thresholded spikes) is superior to low frequency LFP (<4 Hz) in decoding movement (Bansal et al. 2011). However, the frequency range of LFP used by Bansal et al. is different from the frequency range of *lf*-MWP (0–234 Hz) used in our study. In addition, Bansal et al. compared decoding performance for reach and grasp (Bansal et al. 2011), whereas in our study we focused on decoding only isolated hand movements (hand-close, hand-open and wrist movements) with no reaching tasks. Together this can explain the contrasting nature of results obtained in the two studies. In general, diverse range of neural signals corresponding to different frequency ranges recorded from intracortical arrays can provide distinct and complementary information for decoding movement. For example, one study showed that LFP (10–40 Hz) is a reliable indicator of movement onset but doesn’t encode kinematic features, like position, direction, and velocity (Barrese et al. 2013); another study shows that the LFP signal near gamma band could contain information for arm movement direction (Mehring et al. 2004); while LFP from 100 to 300 Hz could encode kinematic information like speed (Bansal et al. 2011). On the other hand, TCs and SUA can better encode information related to movement direction direction (Fraser et al. 2009). Similarly, it has been shown that MUA (300 Hz – 6 kHz) can outperform SUA and LFP for classifying multiple discrete grasp types (Stark and Abeles 2007).

## Conclusion

In conclusion, we give details on a new method to extract MWP-based neural features from chronic human intracortical data. The results presented here show a systematic, long-term tracking of MWP-based neural features and their comparison with traditionally used features such as LFP, MUA and TCs, as well as SVM algorithm-based decoding of complex discrete hand movements from a human brain over 3 years. This information is important and valuable as it will allow researchers to objectively evaluate features of neural data captured in the chronic phase of MEA implantation, which has significance for building and improving upon algorithms in BCI systems. Our results indicate that wavelet processing based neural features engineering can be a viable new way to automate neural signal processing for big data and that *mf*-MWP based neural features can be an optimal signal for BCI decoding applications with stability and performance comparable to traditionally used MUA features. While this report provides important information about signal stability in the human motor cortex and useful comparisons between different features derived from neural signals, our other recent works have shown that this feature engineering and decoding approach can enable real-time control of a BCI-controlled functional electrical stimulation system to enable multiple hand movements in the same study participant.

## Additional files

Additional file 1: **Figure S1.** Temporal corrlation between different paired neural features time series over the course of study. In each insertion, y-axis shows the correlation value range from 0 to 1 and x-axis shows the days post-implantation. Each data point represents the average correlation value of a day between a paired types of neural features from Task 2. The dashed line indicates a 3rd order polynomial fit for the data. Each group of color coded data points shows the temporal correlation of a given paired types of neural features. Taken the first figure as an example, it shows the temporal correlation between the *hf*-MWP feature and other neural features (using *hf*-MWP features as the base time series). (JPG 288 kb)

Additional file 2: **Figure S2.** Overall decoding accuracy affected by using different features averaged from different scales of MWP. Each data point here shows the average of overall decoding accuracy time series over the course of the study for Task2. The error bar indicates the standard deviation of its overall accuracy time series. Selection of MWP features from scales [3, 4, 5, 6] enables the best decoding accuracy, 85.99 ± 4.29%. However, when use MWP from scales [3, 4, 5] and [4, 5, 6], these input features could also enable a very similar level of decoding with overall accuray of 85.81 ± 4.42% and 85.93 ± 4.26%, respectively. One way ANOVA test indicates these three groups of decoding performances were non-significant different (*p* = 0.94, *n* = 128). Using MWP scales with less overlap with scales [3, 4, 5, 6] would induce a larger decrease in decoding accuray. Statistical analysis indicates decoding performances, when using scales within [3, 4, 5, 6] and outside this frequency, were significant different with *p* < 0.001 (*n* = 128). (For frequency bands of each scale in MWP, please refer to Table 1). (JPG 72 kb)

## Abbreviations

BCI: Brain computer interface
FES: Functional electrical stimulation
hf-MWP: High-frequency mean wavelet power
lf-MWP: Low-frequency mean wavelet power
LFP: Local field potential
MEA: Microelectrode array
mf-MWP: Mid-frequency mean wavelet power
MUA: Muti-unit activity
MWP: Mean wavelet power
RMS: Root-mean-square
SCI: Spinal cord injury
SNR: Signal-noise-Ratio
SUA: Single-unit activity
SVM: Support vector machine
TC: Threshold crossing
