# Supplementary figures and images for: Extracting wavelet based neural features from human intracortical recordings for neuroprosthetics applications
Source: Bioelectron Med. 2018 Jul 31;4:11. doi: 10.1186/s42234-018-0011-x (PMC7098253; doi:10.1186/s42234-018-0011-x)

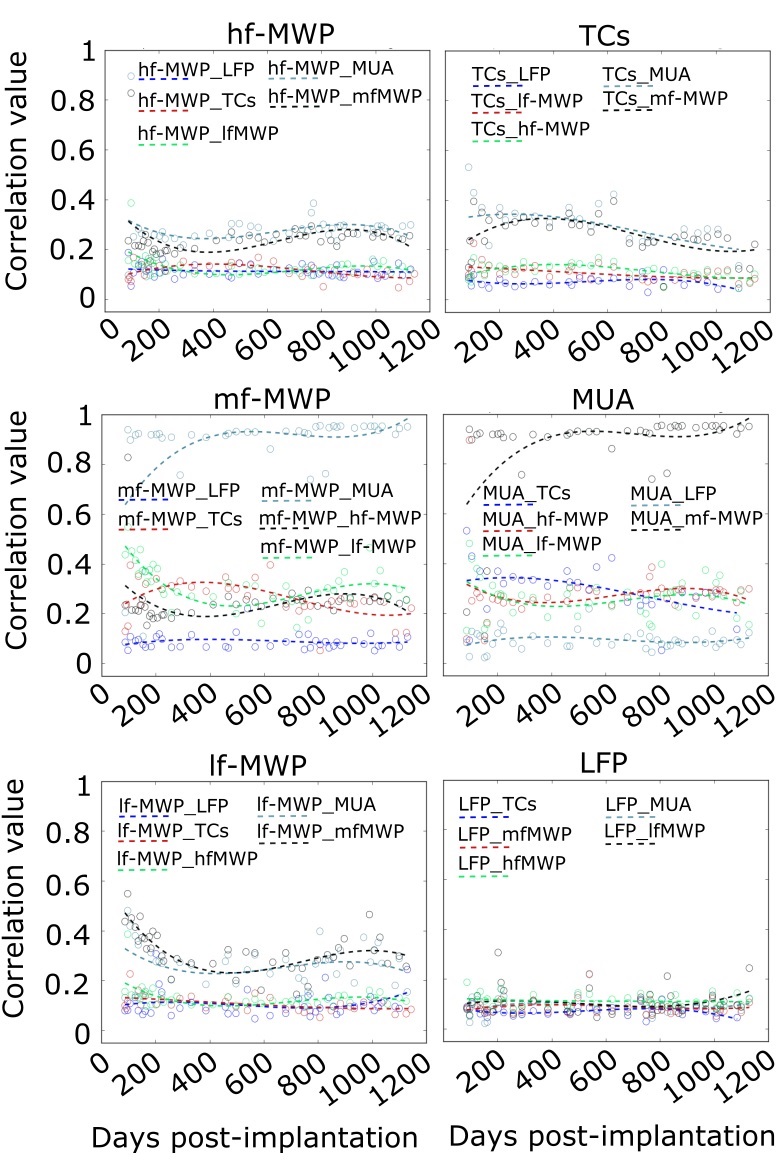

Supplement: Supplementary file 1 — Figure S1. Temporal corrlation between different paired neural features time series over the course of study. In each insertion, y-axis shows the correlation value range from 0 to 1 and x-axis shows the days post-implantation. Each data point represents the average correlation value of a day between a paired types of neural features from Task 2. The dashed line indicates a 3rd order polynomial fit for the data. Each group of color coded data points shows the temporal correlation of a given paired types of neural features. Taken the first figure as an example, it shows the temporal correlation between the hf-MWP feature and other neural features (using hf-MWP features as the base time series). (JPG 288 kb) [file 42234_2018_11_MOESM1_ESM.jpg]

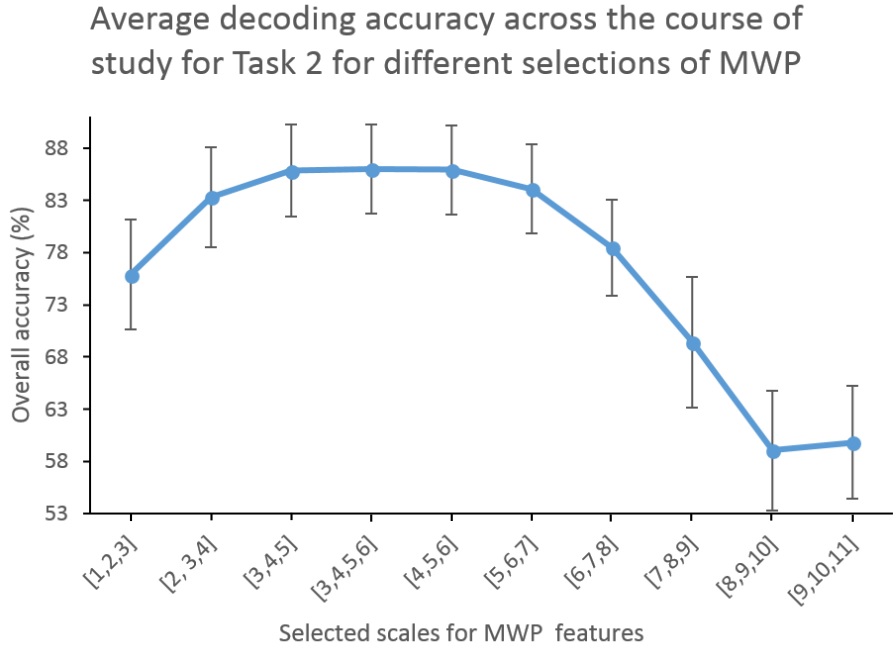

Supplement: Supplementary file 2 — Figure S2. Overall decoding accuracy affected by using different features averaged from different scales of MWP. Each data point here shows the average of overall decoding accuracy time series over the course of the study for Task2. The error bar indicates the standard deviation of its overall accuracy time series. Selection of MWP features from scales [3, 4, 5, 6] enables the best decoding accuracy, 85.99 ± 4.29%. However, when use MWP from scales [3, 4, 5] and [4, 5, 6], these input features could also enable a very similar level of decoding with overall accuray of 85.81 ± 4.42% and 85.93 ± 4.26%, respectively. One way ANOVA test indicates these three groups of decoding performances were non-significant different (p = 0.94, n = 128). Using MWP scales with less overlap with scales [3, 4, 5, 6] would induce a larger decrease in decoding accuray. Statistical analysis indicates decoding performances, when using scales within [3, 4, 5, 6] and outside this frequency, were significant different with p < 0.001 (n = 128). (For frequency bands of each scale in MWP, please refer to Table 1). (JPG 72 kb) [file 42234_2018_11_MOESM2_ESM.jpg]
